# Ferruginol Restores SIRT1-PGC-1α-Mediated Mitochondrial Biogenesis and Fatty Acid Oxidation for the Treatment of DOX-Induced Cardiotoxicity

**DOI:** 10.3389/fphar.2021.773834

**Published:** 2021-11-24

**Authors:** Weili Li, Jing Cao, Xiaoping Wang, Yawen Zhang, Qianbin Sun, Yanyan Jiang, Junkai Yao, Chun Li, Yong Wang, Wei Wang

**Affiliations:** ^1^ School of Life Science, Beijing University of Chinese Medicine, Beijing, China; ^2^ School of Traditional Chinese Medicine, Beijing University of Chinese Medicine, Beijing, China; ^3^ Modern Research Center for Traditional Chinese Medicine, Beijing University of Chinese Medicine, Beijing, China; ^4^ Beijing Key Laboratory of TCM Syndrome and Formula, Beijing, China; ^5^ Key Laboratory of TCM Syndrome and Formula (Beijing University of Chinese Medicine), Ministry of Education, Beijing, China

**Keywords:** ferruginol, mitochondrial biogenesis, fatty acid oxidation, SIRT1, PGC-1α, doxorubicin

## Abstract

**Background:** Doxorubicin (DOX), a broad-spectrum chemotherapy drug, has life-threatening cardiotoxicity. Therefore, searching cardioprotective drugs for DOX-induced cardiotoxicity (DIC) is urgently needed.

**Objectives:** This study aimed to explore cardioprotective effect and specific mechanism by which Ferruginol (FGL) attenuated DIC *in vivo* and *in vitro*.

**Methods:** We evaluated the cardioprotection of FGL and performed high-throughput RNA-Seq on a DIC mouse. Whereafter, multiple methods, including western blot, RT-qPCR, a transmission electron microscope, CO-IP, immunofluorescence, and other staining methods, and antagonist of SIRT1 and PGC-1α were utilized to confirm the cardioprotection and molecular mechanism of FGL.

**Results:** FGL-exerted cardioprotection manifested as enhanced cardiac function and reduced structural damage and apoptosis. The transcriptome and other results revealed that FGL facilitated PGC-1α-mediated mitochondrial biogenesis and fatty acid oxidation (MB and FAO) by increasing the expression of PGC-1α and concurrently promoting the expression of SIRT1-enhancing deacetylase SIRT1 deacetylating and activating PGC-1α.

**Conclusions:** These results documented that FGL exerted cardioprotective effects restoring MB&FAO via the SIRT1–PGC-1α axis.

## Introduction

The anthracycline antibiotic doxorubicin (DOX) is widely used as an effective chemotherapeutic drug for a wide range of cancers (Carter, 1975; Carvalho et al., 2009). However, the dose-dependent cardiotoxicity of DOX severely limits its clinical application. The cardiotoxicity experienced with DOX can range from asymptomatic reductions in the left ventricular ejection fraction to cardiomyopathy, badly myocardial infarction, and congestive heart failure (Shakir and Rasul, 2009; Bernstein and Burridge, 2014). Dexrazoxane is the only cardioprotectant currently approved by the United States Food and Drug Administration for combining with DOX in cancer therapy (Schuchter et al., 2002; Kane et al., 2008). Unfortunately, dexrazoxane is criticized for inducing secondary malignancies and aggravating myelosuppression and has been prohibited in children by the European Medicines Agency in 2011 (Tebbi et al., 2007; European Medicines Agency, 2011). Therefore, a new cardioprotectant for DOX-induced cardiotoxicity (DIC) is urgently needed.

Over the past decades, extensive studies sought to elucidate the mechanistic underpinnings that reveal the pathological occurrence and development of DIC and provide research links for its treatment (Sokolove, 1994; Arola et al., 2000; Minotti et al., 2004). Impressively, abnormalities in mitochondrial functions including defects in the respiratory chain/oxidative phosphorylation (OXPHOS) system, reduction of fatty acid oxidation, mitochondrial DNA damage, and modulation of mitochondrial sirtuin activity have become primary causative factors of DIC (Zhou et al., 2001a; Zhou et al., 2001b; Tokarska-Schlattner et al., 2002). Thus, there is a high level of interest in developing therapeutic strategies aiming at modulating the regulatory pathways that control mitochondrial function.

PGC-1α, as a member of the peroxisome proliferator-activated receptor gamma coactivator-1 (PGC-1) family, plays a major role in transducing and integrating physiological signals governing metabolism, differentiation, and cell growth to the transcriptional machinery controlling mitochondrial biogenesis and function through direct interaction and coactivation with PPARs, ERRs, and NRFs (Rowe et al., 2010; Scarpulla et al., 2012). To date, more reports have verified that PGC-1α is directly deacetylated and reactivated in nuclei by the NAD+-dependent deacetylase SIRT1 in response to changes in energy depletion, thus influencing multiple bioprocesses mediated by PGC-1α (Lagouge et al., 2006; Gerhart-Hines et al., 2007; Jeninga et al., 2010). Therefore, we believe that DOX causes myocardial energy deficiency and alteration of the SIRT1-PGC-1α regulatory signal, which may be major pathogenic mechanisms of DIC, and also a potential druggable pathway to coordinate mitochondria-related bioprocesses for DIC treatment.

Ferruginol, a natural polyphenol and terpenoid isolated from Salvia plants, has demonstrated a variety of pharmacological potentials including antioxidant, antitumor, gastroprotective, and neuroprotective activity (Rodríguez et al., 2006; Bispo de Jesus et al., 2008; Saijo et al., 2015; Zolezzi et al., 2018). However, there were very little evidences for cardioprotective activity of FGL. Our study for the first time demonstrated strong protective effect of FGL on DIC mouse heart and H9C2 cells. Thus, we hypothesized that FGL alleviated DIC by SIRT1–PGC-1α-mediated myocardial MB&FAO, and above, the pharmacological effects and mechanisms of FGL were verified *in vivo* and *in vitro*.

**GRAPHICAL ABSTRACT:**
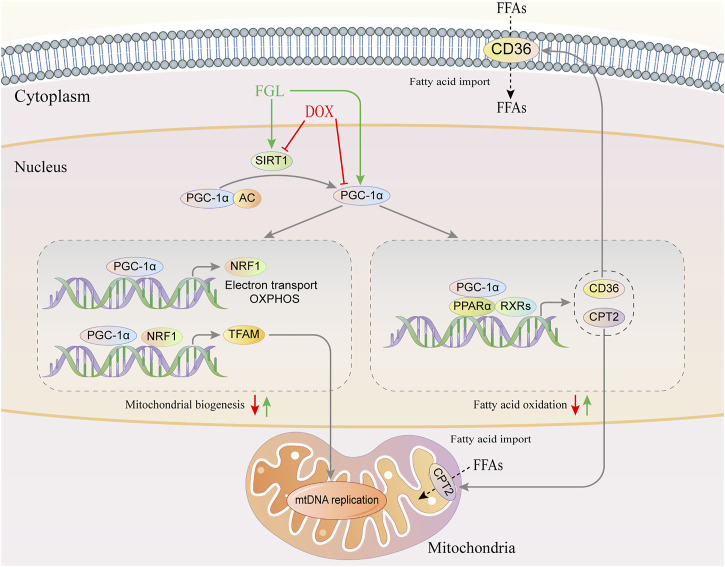
Graphical abstract of the pathogenic mechanism of DOX and the cardioprotective mechanism of FGL.

## Methods

### Materials

All reagents and materials used in this research are documented in the Supplementary Material.

### Construction of DIC Mouse Model and Pharmacological Treatments

Male C57BL/6 mice (20 ± 2 g, 8–10 weeks old) were purchased from Beijing SPF Biotechnology (Beijing, China). All procedures were performed following the guidelines of the Institutional Animal Care and Use Committee and approved by the Beijing University of Chinese Medicine Animal Care Committee. The animals were housed in a 12:12-h light–dark cycle and temperature-controlled (22°C) environment and fed with standard rodent chow and tap water. A DIC model was generated as described previously (Wang et al., 2019; Wang et al., 2020). After a week of adjustable feeding, mice randomly assigned in the model, FGL, and ENA groups were injected by tail vein with DOX following a dose of 5 mg/kg once weekly for 4 weeks. Meanwhile, mice in the control group were treated with saline solution (0.9% NaCl) instead. Then, FGL at 20 mg/kg and ENA at 15 mg/kg were administered intragastrically and daily for 4 weeks at 1 week after last injection of DOX, respectively. The control and model groups also received the same volume of ultrapure water. ENA has been repeatedly reported to protect against DIC in clinical and preclinical studies (Hullin et al., 2018); thus, we applied ENA as a positive control for animal experiments.

### RNA Sequencing and Analysis

Samples were prepared according to the protocol of the manufacturer and then underwent RNA extraction, reverse transcription, and DNA amplification, followed by sequencing on an Illumina HiSeq. We identified differentially expressed genes (DEGs) based on the following criteria: *p* < 0.05 and absolute value of log2fold-change>0. Biological and pathway analysis of DEGs was explored by GO term enrichment analysis and KEGG pathway enrichment analysis, respectively. Protein–protein interaction (PPI) network analysis of DEGs was acquired from the Search Tool for the Retrieval of Interacting Genes (STRING) database (version 11.0; https://www.string-db.org/).

### Echocardiographic Assessment of Cardiac Functions

After 4 weeks’ administration, the mice were immobilized and anesthetized with isoflurane gas for echocardiography. The transthoracic echocardiography was performed in M-mode with the VEVO 2100 echocardiography system (Visual Sonics, Toronto, ON, Canada) by the same operator on all mice. Left ventricular end-systolic volume (LVESV), left ventricular end-diastolic volume (LVEDV), left ventricular end-diastolic dimension (LVEDD), and left ventricular end-systolic dimension (LVESD) were measured using computer algorithms for at least three uninterrupted cardiac cycles. The calculated EF and FS values of the mice treated by DOX were uniformly decreased compared to those in the control group.

### Histological Examination

Cardiac tissue samples were immersed in 4% paraformaldehyde and then embedded in paraffin and cut into 4-μm serial sections. The sections were stained with hematoxylin–eosin (H&E) and Masson trichromatic solution and were then observed under an optical microscope at ×400 magnification.

### Assessment of Serum Biomarkers

Blood samples were obtained from abdominal aorta, and the sera were isolated to measure tissue injury markers, lactate dehydrogenase (LDH), and creatine kinase isoenzymes (CK-MB) with standard diagnostic kits (Nanjing Jiancheng Bioengineering Institute, Jiangsu, China).

### Cell Culture and Pharmacological Treatments

H9C2, HepG2, and MCF-7 cells were grown in DMEM with 10% fetal bovine serum supplement and incubated in humidified air (5% CO_2_) at 37°C. H9C2, HepG2, and MCF-7 cells were grown on 96-well plates at a density of 4 × 104 cells/ml for 24 h and then divided into the control, DOX, and 0.1–50 µM FGL groups. Next, FGL groups were pretreated with FGL, followed by cotreating with 1 µM DOX as previously confirmed (Wang et al., 2020) and FGL (0.1–50 µM) in FGL groups while merely with 1 µM DOX in the DOX group for 24 h. Subsequently, cell viability was detected using 10% CCK-8 (Dojindo, Kumamoto, Japan). H9C2 cells were divided into five groups as follows: control group, DOX group, DOX + FGL, DOX + FGL + Selisistat, and DOX + FGL + SR-18292. Selisistat is an inhibitor of SIRT1, inhibiting the deacetylation activity of SIRT1. SR-18292 is an inhibitor of PGC-1α, promoting the acetylation of PGC-1α and inhibiting its activity as a transcriptional coactivator. Amounts of 1 μM DOX, 0.1 μM FGL, 20 μM Selisistat, and 20 μM SR-18292 were applied, respectively.

### Detection of Apoptosis

The apoptosis of cardiac tissue was determined by terminal deoxynucleotidyl transferase-mediated nick end labeling (TUNEL) according to the instructions of the manufacturer and then staining with DAPI to label the nuclei. In addition, H9C2 cells were incubated with 2 mg/ml Hoechst 33,342 for 30 min at 37°C in the dark for detecting the apoptosis of H9C2 cells. Finally, a fluorescence microscope was used to visualize apoptotic cells.

### Transmission Electron Microscopy

The process was performed as described previously (Wang et al., 2019). Images were acquired under an electron microscope (Leica, Buffalo Grove, IL, United States). Mitochondria were counted randomly based on six images from various fields of view (×2,500 magnification).

### Mitochondrial Membrane Potential

Changes of mitochondrial membrane potential (ΔѰm) were detected using the fluorescent probe JC-1 (Beijing Solaibao Technology Co., Ltd., Beijing, China) according to the user manual. JC-1 is a cationic dye that accumulates in the mitochondria in a potential-sensitive manner. At high ΔΨm, JC-1 accumulates in the mitochondria forming J-aggregates and emitting red fluorescence; at low ΔѰm, the J-monomers generate green fluorescence. After pharmacological treatments, the cells were incubated with 1×JC-1 working solution for 30 min and washed twice with JC-1 dye buffer. Finally, the images were monitored under a fluorescence microscope. ΔΨm was evaluated and calculated by the ratio of red/green fluorescence intensities.

### ROS Detection

DCFH-DA staining was performed for the detection of ROS in H9C2 cells. Pretreated H9C2 cells were cultured with 10 μM DCFH-DA for 30 min at 37°C in the dark. Then DAPI was used to stain the nuclei. The images were acquired using a laser scanning confocal microscope at ×100 magnification.

### Adenosine Triphosphate Content

ATP levels in H9C2 cells were detected by using an ATP assay kit (Beyotime Biotechnology, Shanghai, China). Briefly, H9C2 cells were lysed and centrifuged, and the supernatant was collected for the determination of ATP and total protein content. The content of ATP was normalized to the cellular protein concentration.

### Real-Time Quantitative PCR for RNA Expression Analysis

The process was performed as described previously (Wang et al., 2019). The primers used in this study are shown in Supplementary Material.

### Immunofluorescence Staining

H9C2 cells were fixed with 4% formaldehyde, washed twice with PBS, and then permeabilized with 0.3% Triton X-100. After being blocked with 5% bovine serum albumin, cells were incubated with primary antibodies against SIRT1 and PGC-1α, and then with the corresponding secondary antibodies. The nuclei were counterstained with DAPI. Images were captured by a laser scanning confocal microscope at 1,000× magnification.

### Co-Immunoprecipitation Assay

Co-IP was executed according to the indicated procedures of the manufacturer (Cell Signaling Technology, United States). H9C2 cells were lysed in a lysis buffer. Equivalent proteins from each sample were combined with anti-PGC-1α antibody or control IgG antibody overnight at 4°C. Later, the resulting immunocomplex was precipitated employing Protein G magnetic beads, followed by washing five times. Thereafter, precipitates mixed in the SDS buffer solution were analyzed by western blot with either anti-acetylated lysine antibody to determine the extent of PGC-1α acetylation or PGC-1α antibody to determine the total amount of PGC-1α.

### Western Blotting Analysis

To detect protein expression, western blotting was carried out as previously described (Wang et al., 2019; Wang et al., 2020). The antibodies used in the study are shown in Supplementary Material.

### Statistical Analysis

Statistical analysis was performed using the GraphPad software 6. Numerical results are displayed as mean ± s.d. Statistical significance was evaluated by applying two-tailed unpaired Student’s t-test. Alternatively, comparisons of more than two groups were performed by one-way analysis of variance (ANOVA) with Bonferroni *post hoc* tests for multiple groups to calculate the differences. A *p*-value of <0.05 was considered as statistically significant.

## Results

### Ferruginol Relieved Doxorubicin-Induced Cardiac Structural and Functional Lesion

**FIGURE 1:**
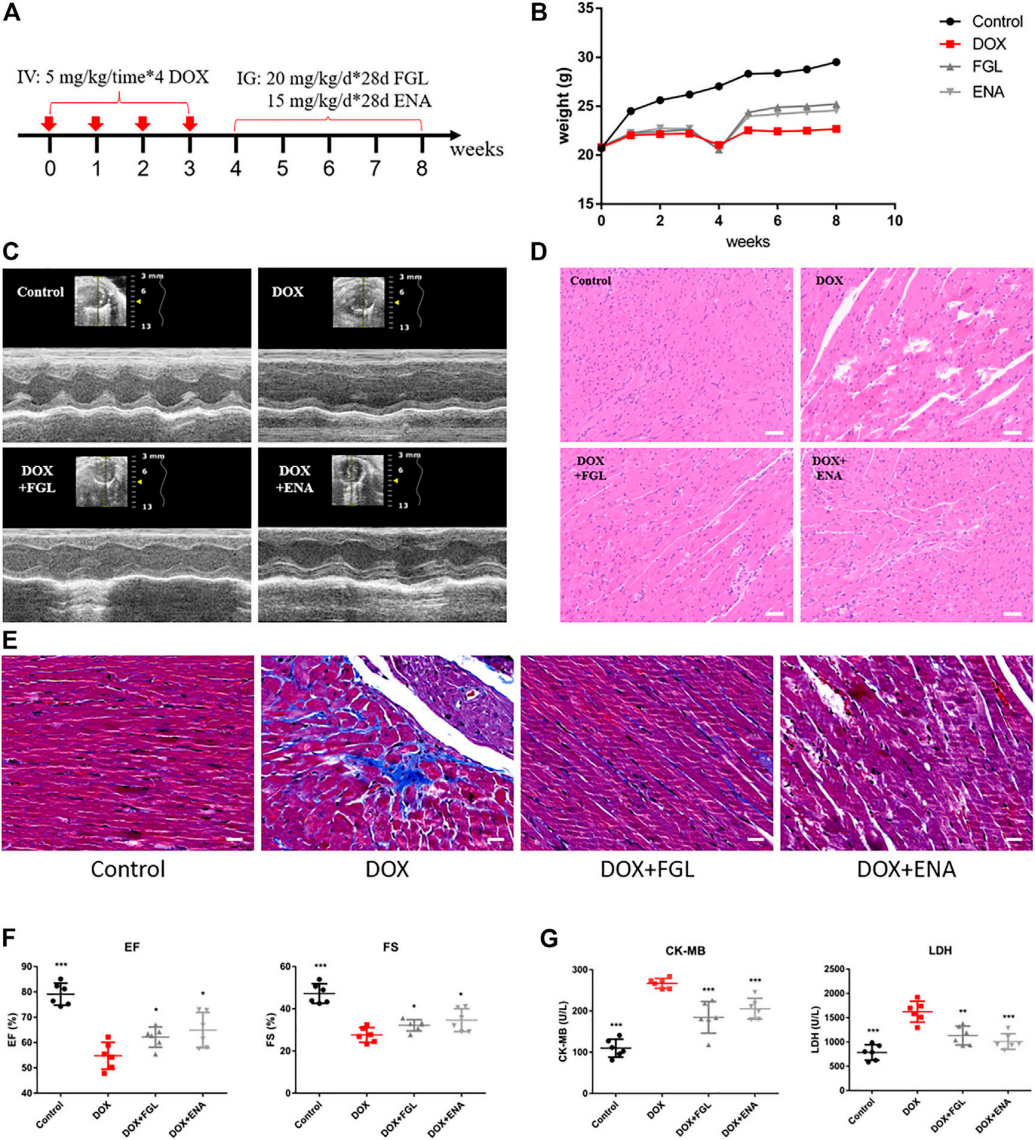
FGL relieved DOX-induced cardiac structural and functional lesion. **(A)** A schematic diagram of animal model generation and drug administration. **(B)** Body weight changes in various groups (n = 10). **(C)** Representative images of echocardiograms. **(D)** Representative images of morphological and structural changes in the myocardium. Scale bar: 50 μm. **(E)** Myocardial fibrosis detection by Masson staining. **(F)** The effects of FGL and ENA on DOX-induced cardiac EF and FS (n = 6). **(G)** The effects of FGL and ENA on cardiac serum CK-MB and LDH (n = 6). *p < 0.05, **p < 0.01, ***p < 0.001 vs DOX group.

The design of animal experiments, including the generation of a DIC model and subsequent pharmacological treatment, is shown in Figure 1A. The body weight of mice was recorded throughout the animal experiment (Figure 1B). Comparison with the control group indicated an evident decline in the body weight in the DOX-treated group. Meanwhile, the body weight of mice in the FGL and ENA groups was significantly increased during weeks 5–8 compared with that in the model group. The mice underwent echocardiography to evaluate cardiac function after 4 weeks of pharmacological intervention. FGL alleviated the decrease in EF and FS values caused by DOX (Figures 1C,F). Consistently, H&E and Masson staining of heart sections showed that DOX induced myofibrillar loss, disordered arrangement, enlarged intercellular structures, and plasma-dissolved cardiomyocytes and myocardial fibrosis, which could be attenuated by FGL (Figures 1D,E). Additionally, increased serum content of LDH and CK-MB, markers of myocardial damage, was observed in the DOX group, while FGL treatment partially reversed these changes (Figure 1G).

### Doxorubicin-Induced Deficiency of Mitochondrial Biogenesis and Fatty Acid Oxidation Mediated by Proliferator-Activated Receptor Gamma Coactivator-1 Were Significantly Improved by Ferruginol

**FIGURE 2:**
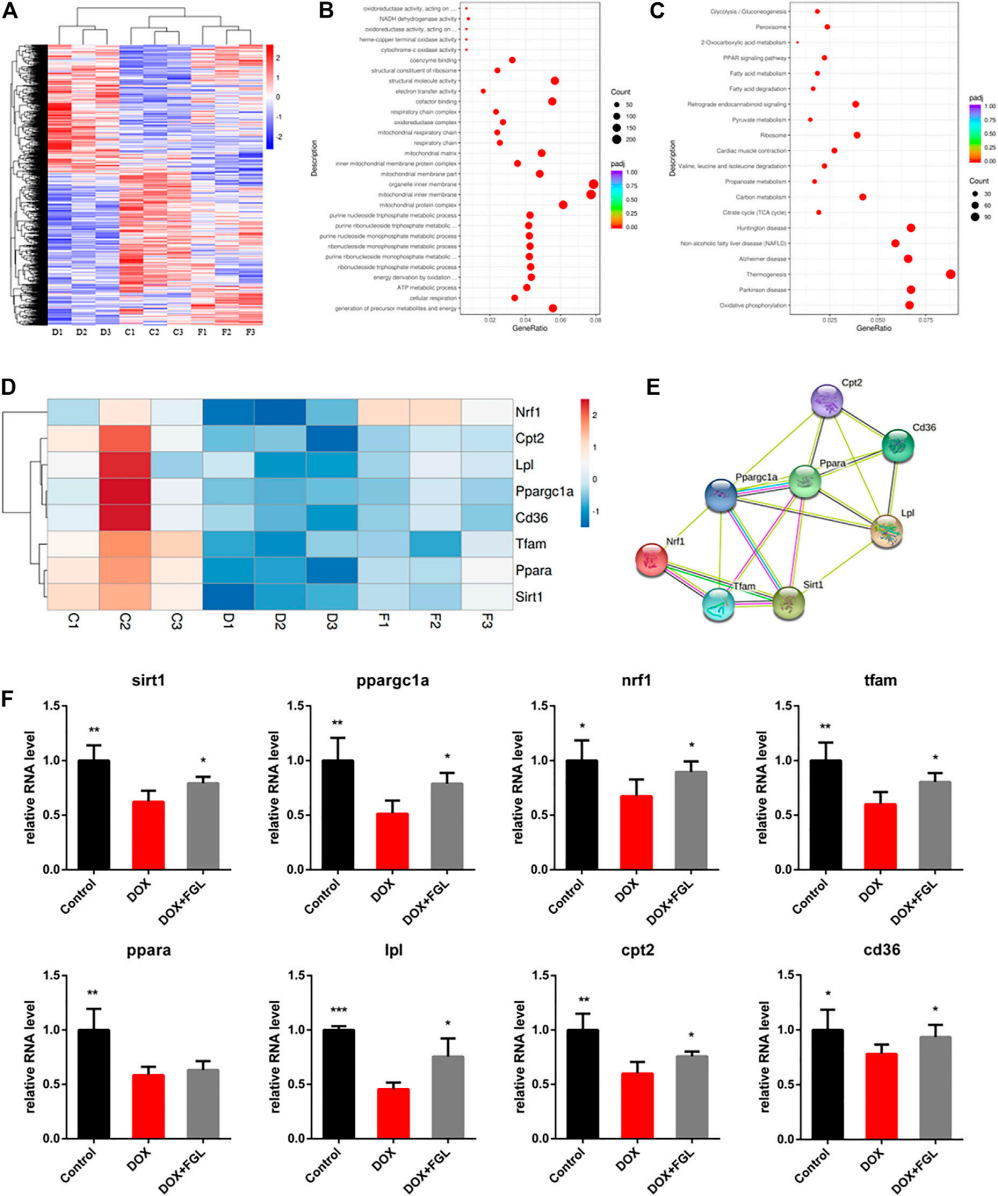
The results of mRNA sequencing and analysis. **(A)** Hierarchical clustering of proteins and samples. **(B, C)** GO and KEGG enrichment analysis between the control and DOX groups. **(D, E)** Representative gene heatmap and PPI diagram associated with MB&FAO. **(F)** Quantitative analysis of representative mRNA associated with MB&FAO by RT-qPCR. (n = 3–5). *p < 0.05, **p < 0.01, ***p < 0.001 vs DOX group.

To further explore the pathogenic mechanism of DOX and underlying molecular mechanisms of FGL on cardiac protection, we performed high-throughput RNA-Seq to investigate DEGs in the control, DOX, and DOX + FGL groups of mice. Figure 2A showed distinct hierarchical clustering of the genes and groups. Gene ontology and KEGG analysis between the control and DOX groups revealed that genes related to mitochondria/metabolism, fatty acid oxidation, PPAR signal pathway, etc. were significantly enriched (Figures 2B,C). Then we presented eight DEGs involved in mitochondrial metabolism, biogenesis, and PPAR signaling pathways between the control and DOX groups in the form of heatmap (Figure 2D). Protein–protein interaction (PPI) network analysis (version 11.0; https://www.string-db.org/) indicated that a cluster containing eight nodes (Sirt1, Ppargc1a, Ppara, Nrf1, Tfam, Cd36, Lpl, and Cpt2) possessed a strong connection (Figure 2E).

To validate the results of RNA-Seq and quantify the expression of these key genes, we assessed eight DEGs involved in mitochondrial biogenesis and the PPAR signaling pathway by quantitative RT-PCR on three to five heart samples. The results of the RT-qPCR analysis were in good agreement with the RNA-Seq data that DOX resulted in the downregulation of eight genes compared with the control group, while FGL significantly increased the RNA levels of all genes except Ppara compared with the DOX group (Figure 2F).

### Ferruginol Inhibited Doxorubicin-Induced Apoptosis and Oxidative Injury via SIRT1 and Proliferator-Activated Receptor Gamma Coactivator-1 *In Vivo* and *In Vitro*

**FIGURE 3:**
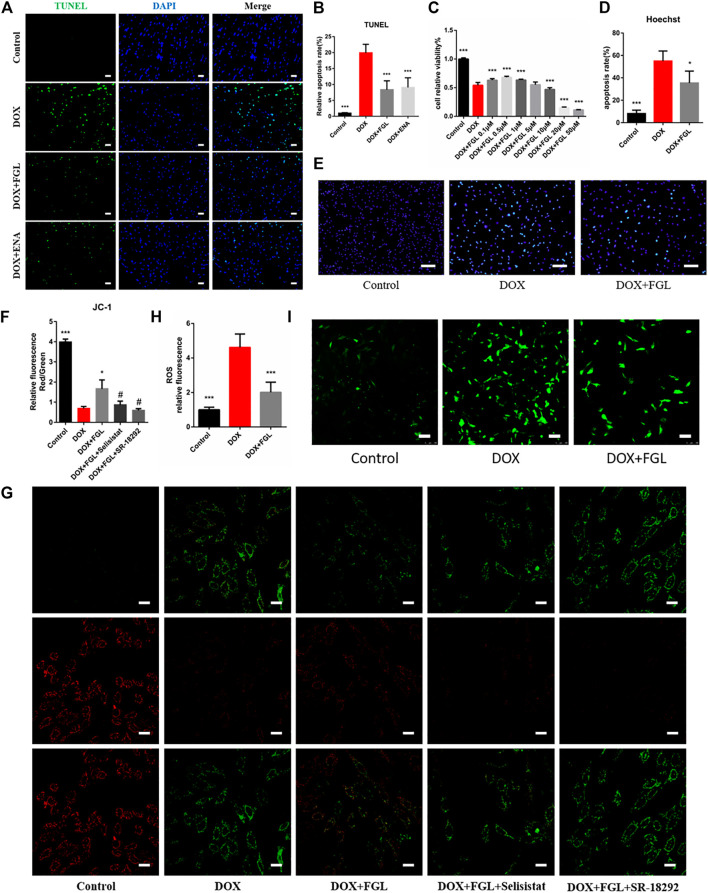
FGL inhibited DOX-induced apoptosis and oxidative injury *in vivo* and *in vitro*. **(A, B)** Representative images and quantitative analysis of apoptosis by TUNEL in the mouse myocardium (n = 6). Scale bar: 20 μm. **(C)** Effective concentration of FGL in H9C2 cells (n = 6). **(D, E)** Representative images and quantitative analysis of apoptosis by Hoechst staining in H9C2 cells (n = 5). Scale bar: 100 μm. **(F, G)** Representative images and quantitative analysis of mitochondrial membrane potential measured by JC-1 staining in H9C2 cells (n = 3). Scale bar: 25 μm. **(H, I)** Representative images and quantitative analysis of ROS by DCFH-DA staining in H9C2 cells (n = 6). Scale bar: 100 μm *p < 0.05, ***p < 0.001 vs DOX group. #*p* < 0.05 vs. (DOX + FGL) group.

DOX-induced apoptosis has long been considered to be one of the leading causes of cardiac function decline. Therefore, we evaluated the apoptosis of cardiomyocytes *in vivo* and *in vitro*. As shown in Figure 3C, the minimum concentration of FGL that enhanced cell viability was 0.1 μM. TUNEL staining of mouse heart slices and Hoechst staining of H9C2 cells showed that FGL alleviated DOX-induced apoptosis (Figures 3A,B,D,E). Moreover, mitochondrial membrane potential reduction is a hallmark event of early apoptosis. The results showed that DOX caused the notable drop of the mitochondrial membrane potential, which could be alleviated by FGL. Compared with the FGL-treatment group, selisistat and SR-18292 reduced the mitochondrial membrane potential (Figures 3F,G). We also found that DOX caused a large amount of ROS production, while FGL significantly reduced ROS production. These results suggested that FGL alleviated apoptosis and ROS production by associating with SIRT1 and PGC-1α.

### Ferruginol Promoted Mitochondrial Biogenesis and Fatty Acid Oxidization *In Vivo*

**FIGURE 4:**
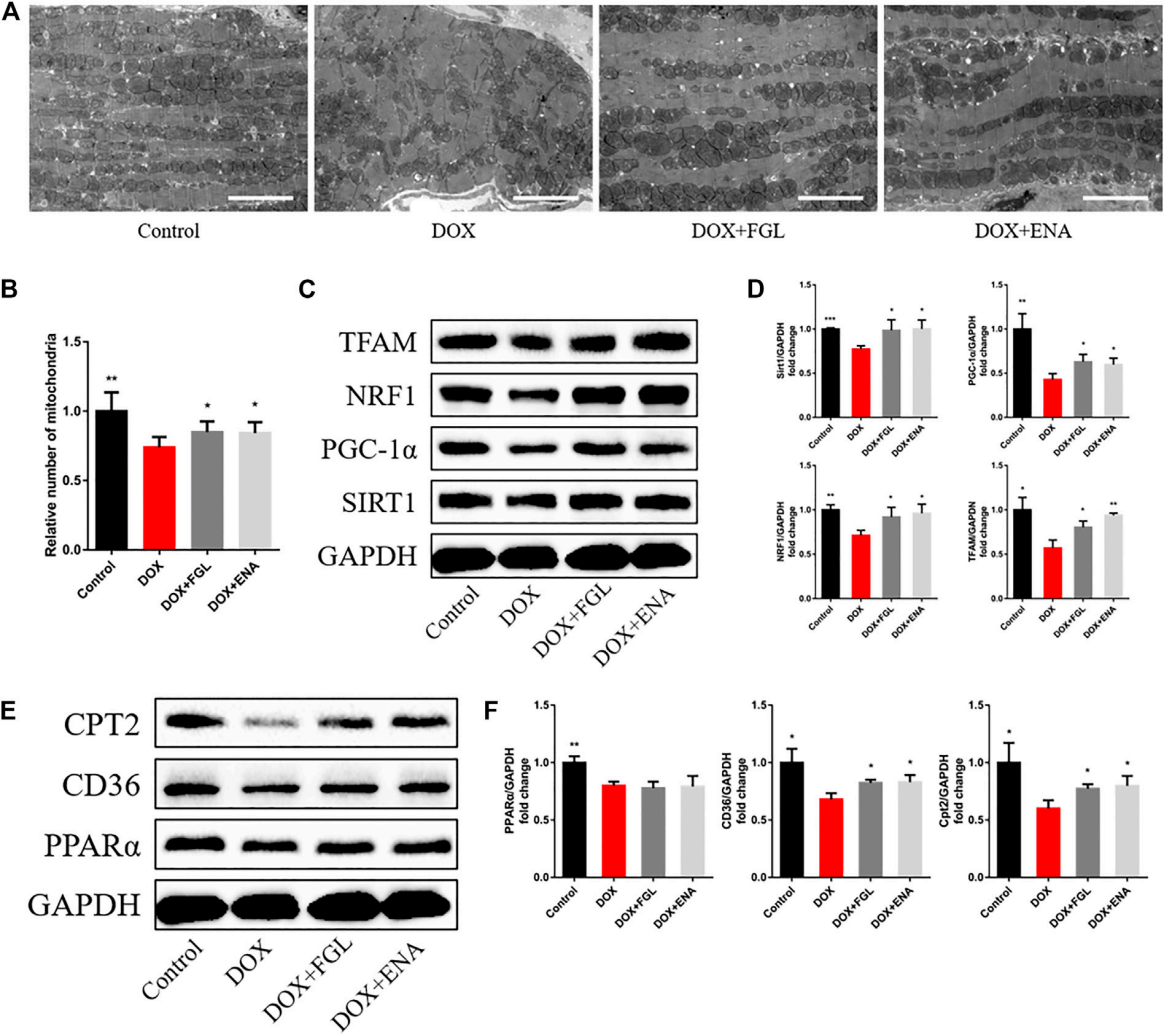
FGL promoted mitochondrial biogenesis and fatty acid oxidization *in vivo*. **(A, B)** Representative images and quantitative analysis of electron micrographs of the heart (n = 6). Scale bar: 5.0 μm. **(C–F)** Expression levels of mitochondrial biogenesis and fatty acid oxidization-associated proteins were quantified and shown as relative protein expression levels after normalization to GAPDH (n = 3–5). *p < 0.05, **p < 0.01, ***p < 0.001 vs DOX group.

We then hypothesized that FGL exerted cardioprotection by improving mitochondrial biogenesis and fatty acid oxidization. In DIC mice, we found that DOX led to loose arrangement of mitochondria and a large reduction in the number of mitochondria via TEM, while FGL relieved the above adverse changes (Figures 4A,B). Moreover, coinciding with the results of RNA-seq, the expressions of mitochondrial biogenesis and fatty acid oxidization markers were decreased in the DOX group compared to those in the control group, and these changes were relieved by FGL (Figures 4C–F). However, DOX-induced reduction in the protein levels of PPARα was not mitigated by FGL. PGC-1α acts as a transcriptional coactivator to promote the transcriptional regulation of PPARα to downstream proteins (CD36, CPT2, LPL, etc.). We concluded that FGL merely upregulated the expression of PGC1-α without improving the expression of PPARα and thus activated the transcriptional activity of PPARα to promote the expression of downstream genes. In addition, the expression of SIRT1, a deacetylase, was also significantly enhanced by FGL treatment. Combined with reported research that SIRT1 deacetylated PGC-1α and activated its transcriptional activity, we proposed that FGL may promote downstream MB&FAO through the SIRT1–PGC-1α axis.

### Ferruginol Facilitated Mitochondrial Biogenesis and Fatty Acid Oxidization via SIRT1-Proliferator-Activated Receptor Gamma Coactivator-1 Axis *In Vitro*

**FIGURE 5:**
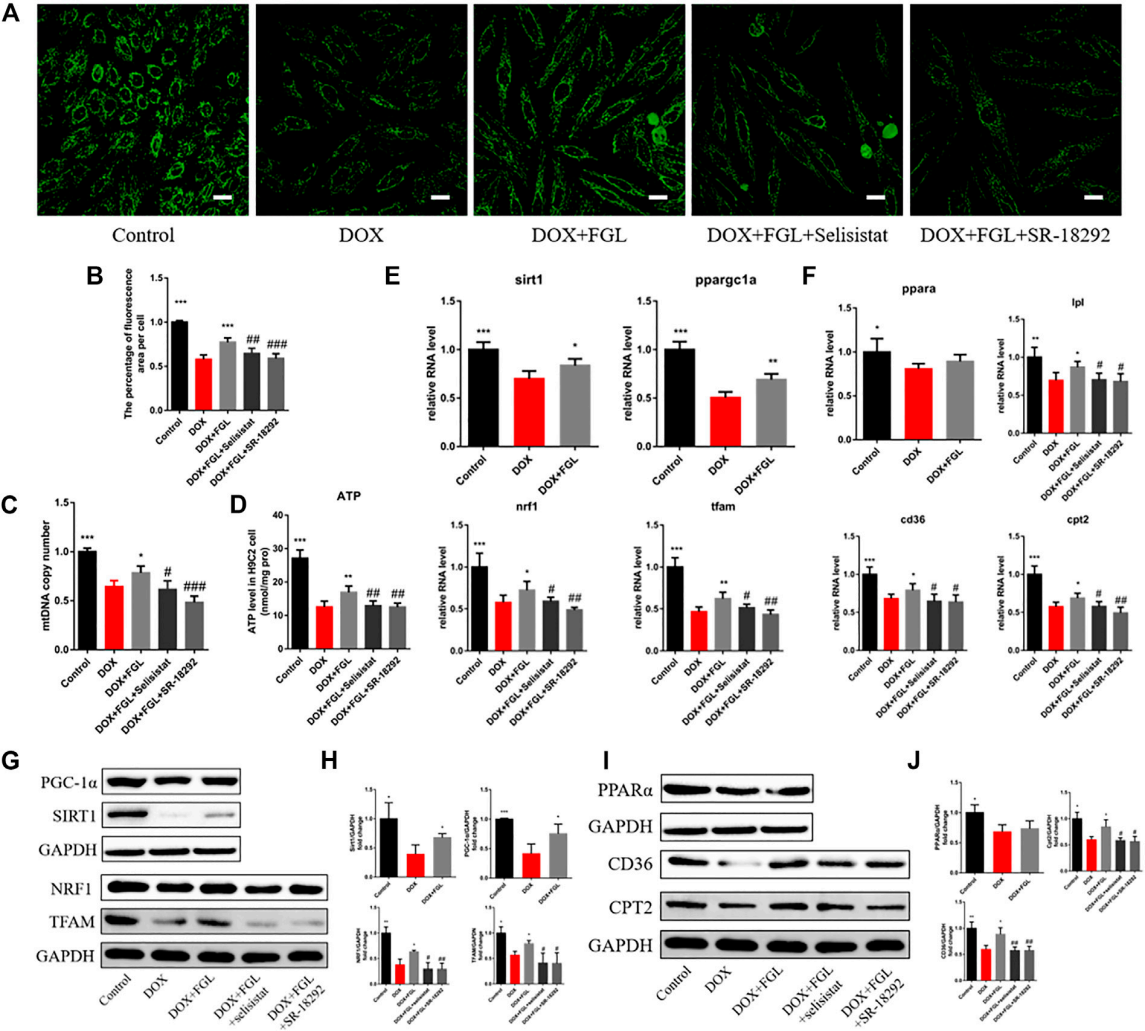
FGL promoted mitochondrial biogenesis and fatty acid oxidization *in vitro*. **(A, B)** Representative images and quantitative analysis of the number of mitochondria by mitotracker staining in H9C2 cells (n = 6). Scale bar: 25 μm. **(C)** The assessment of the mtDNA copy number in H9C2 cells by RT-qPCR (n = 4). **(D)** The content of ATP in H9C2 cells (n = 5). **(E, F)** The expression level of mitochondrial biogenesis and fatty acid oxidization-associated mRNA in H9C2 cells quantified and shown as relative RNA levels after normalization to GAPDH (n = 5). **(G–J)** The expression level of mitochondrial biogenesis and fatty acid oxidization-associated proteins in H9C2 cells quantified and shown as relative protein expression levels after normalization to GAPDH (n = 3–5). *p < 0.05, **p < 0.01, ***p < 0.001 vs DOX group. #*p* < 0.05, ##*p* < 0.01, ###*p* < 0.001 vs. (DOX + FGL) group.

To further confirm whether FGL facilitated MB&FAO through the SIRT1–PGC-1α pathway, we cotreated FGL with selisistat and SR-18292 *in vitro*, respectively. First, we found that DOX resulted in a decrease in the number of mitochondria per unit area *in vitro* consistent with the results of electron microscopy *in vivo*, mtDNA copy number, and ATP content *in vitro*. Contrariwise, FGL contributed to a marked increase in these aspects compared to the DOX group, which were significantly reversed by selisistat and SR-18292 (Figures 5A–D). Impressively, we found that FGL inhibited the downregulation of MB&FAO marker except PPARα induced by DOX; however, both selisistat and SR-18292 significantly reversed the effects of FGL on the downstream mitochondrial biogenesis marker (Nrf1 and TFAM) and the fatty acid oxidation marker (CD36, CPT2, and LPL) (Figures 5E–J). These collective data documented that FGL coordinated MB&FAO through SIRT1 and PGC-1α. However, its specific molecular mechanism between SIRT1 and PGC-1α still needs to be further confirmed.

### Ferruginol Promoted the Deacetylation of Proliferator-Activated Receptor Gamma Coactivator-1 by SIRT1

**FIGURE 6:**
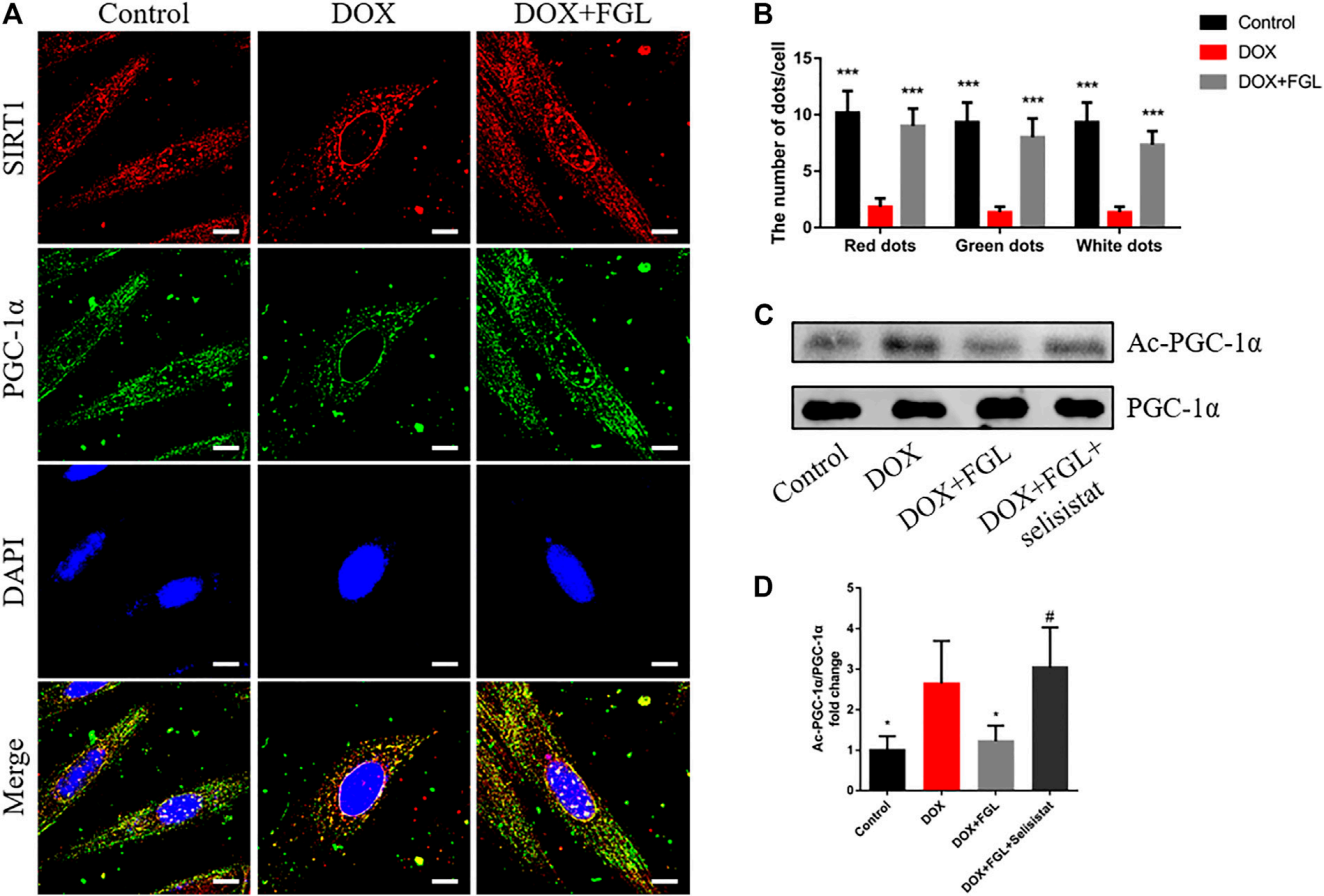
FGL promoted the deacetylation of SIRT1 to PGC-1α *in vitro*. **(A, B)** Representative images and quantitative analysis of SIRT1 and PGC-1α and their colocalization in the nucleus (n = 6). Scale bar: 10 μm. **(C, D)** Representative bands and the ratio of acetylated PGC-1α to total PGC-1α detected by CO-IP of PGC-1α and acetylated lysine (n = 4). *p < 0.05, **p < 0.01, ***p < 0.001 vs DOX group. #*p* < 0.05 vs. (DOX + FGL) group.

In order to confirm the specific interaction between SIRT1 and PGC-1α and whether FGL could interfere with this interaction, the binding of SIRT1 and PGC-1α and the acetylation level of PGC-1α were detected by immunocoprecipitation and immunofluorescence. As shown in Figure 6A, SIRT1 and PGC-1α were distributed in both the cytoplasm and the nucleus, and both of them showed obvious fluorescent spots in the nucleus. Compared with the control group, in the nucleus, DOX resulted in a significant reduction of SIRT1 (red) and PGC-1α (green) and a decrease in colocalization (white). Compared with the DOX group, FGL increased the number of SIRT1 and PGC-1α fluorescence spots and colocalization of both (Figures 6A,B). These results revealed that DOX treatment reduced the expression and binding of SIRT1 and PGC-1α in the nucleus, while FGL promoted the expression and binding of SIRT1 and PGC-1α in the nucleus. The results of CO-IP showed that FGL inhibited the increased acetylation of PGC-1α induced by DOX (Figures 6C,D), while selisistat increased the acetylation of PGC-1α. Collectively, we concluded that FGL inhibited the acetylation of PGC-1α by promoting the expression of SIRT1 and, concurrently, increased the expression of PGC-1α to facilitate PGC-1α-mediated MB&FAO.

### Ferruginol Did Not Interfere With Antitumor Effect of Doxorubicin

**FIGURE 7:**
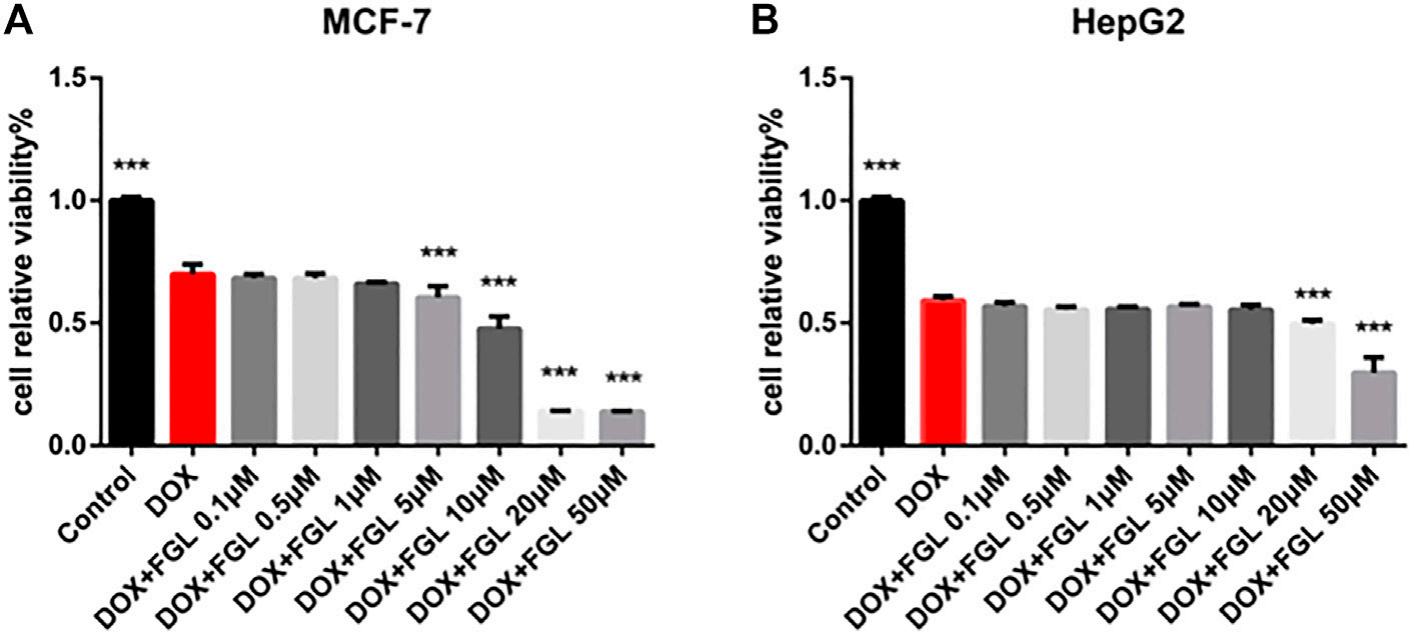
FGL did not interfere with the antitumor effect of DOX. **(A, B)** The influence of FGL on the anticancer effect of DOX on MCF-7 and HepG2 cells was determined by CCK-8 assay (n = 6). ***p < 0.001 vs DOX group.

According to current research on DIC, identification of cardioprotective agents without interfering with the anticancer effect of DOX has been of interest to oncologists and cardiologists. Thus, in addition to confirming the protective effect of FGL on cardiomyocytes, we evaluated whether FGL interferes with the anticancer effect of DOX on the human breast carcinoma cell line MCF-7 and human hepatocellular carcinoma cell line HepG2. As shown in Figure 7, FGL within 0.1–1 μM did not affect the inhibition of cancer cell viability by DOX (Figures 7A,B).

## Discussion

Doxorubicin is a first-line anthracycline quinone and effective anticancer drug extensively used for the treatment of solid and hematologic malignancies (Carter, 1975; Carvalho et al., 2009). However, its clinical use is evidently impeded by life-threatening cardiotoxicity that eventually causes dilated cardiomyopathy and heart failure (McGowan et al., 2017; Mancilla et al., 2019). To date, dexrazoxane is the only FDA-approved cardioprotectant for the prevention of DIC (Schuchter et al., 2002; Kane et al., 2008). However, its use in children was contraindicated by the European Medicines Agency in 2011 (Tebbi et al., 2007; European Medicines Agency, 2011) because of concerns of growing incidence of infection, myelosuppression, second primary malignancies, tumor-killing capacity reduction, and its unestablished efficacy in children. Accordingly, developing new, safe, and effective cardioprotectant to minimize DOX-induced cardiotoxicity is of great significance. For the first time, our study discovered the impressively cardioprotective effect and comprehensively documented the mechanism of FGL against DIC.

Mitochondrial damage events including deficiency of mitochondrial biogenesis and OXPHOS system, reduction of fatty acid oxidation, and ATP production and DNA damage were identified as the leading cause of DIC by most studies (Zhang et al., 2012; Carvalho et al., 2014; Osataphan et al., 2020). The deterioration of the mitochondrial function resulting from DOX eventually drives apoptosis, necrotic cell death, etc., which is fatal to energy-hungry heart with negligible regeneration and leads to subsequent life-threatening left ventricular dysfunction, cardiomyopathy, and heart failure (Felker et al., 2000; Cardinale et al., 2015). Consistently, GO and KEGG analysis of our transcriptome results displayed that DOX-induced DEGs were significantly enriched in mitochondria-associated bioprocesses and signaling pathways compared with the control group. These findings positively suggest that mitochondrial protection strategies should be proposed as potential solutions. Enlighteningly, our study found that FGL well alleviated DOX-induced cardiac structural and functional damage and deficiency of MB&FAO, which suggested that FGL targeted MB&FAO to protect against DIC.

PGC-1α is a transcriptional coactivator that modulates MB and FAO. In particular, PGC-1α dramatically induces the gene expression of NRF1 and physically interacts with Nrf1 and coactivates its transcriptional activity on TFAM, a key transcriptional activator that translocates to mitochondria and activates mitochondrial DNA replication and transcription (Lin et al., 2005). From another aspect, PGC-1 binds to and coactivates PPARα, thereby inducing numerous genes crucial for fatty acid metabolism, including CD36, CPT2, and lipidolysis LPL (Burkart et al., 2007; Pawlak et al., 2015). Both reported studies and our results demonstrated that DOX undermined the PGC-1α signaling pathway resulting in dysfunctional mitochondrial biogenesis, thus triggering a cardiac injury (Guo et al., 2014; Du et al., 2019). Thus, PGC-1α is promising to be a target for the treatment of DIC. So far, accumulating studies reported that PGC-1α is significantly regulated posttranslationally by phosphorylation, acetylation, and methylation (Puigserver et al., 2001; Rodgers et al., 2005; Li et al., 2007), among which deacetylation and reactivation of PGC-1α mediated by NAD+-dependent SIRT1 deacetylase have gradually attracted the attention of cardiologists.

Based on the analysis of transcriptome results, experimental verification, and literature review, we hypothesized that FGL protected against DIC by promoting MB&FAO through the SIRT1–PGC-1α axis. The results demonstrated that FGL increased the number of mitochondria, the mtDNA copy number, the mitochondrial membrane potential, the ATP production, and the mRNA and protein expression of MB&FAO markers, while selisistat and SR-18292 significantly reversed these changes. More specifically, FGL promoted the binding of SIRT1 and PGC-1α and deacetylation of PGC-1α, while selisistat inhibited the deacetylation activity of SIRT1 and strikingly enhanced the acetylation level of PGC-1α. In addition, we found that FGL promoted the expression of SIRT1 and enhanced the deacetylation and transcriptional coactivator activity of PGC-1α without affecting the expression of PPARα. This indicated that FGL promotes the expression of downstream genes of PPARα through the SIRT1–PGC-1α axis instead of PPARα.

In conclusion, our study innovatively found the cardioprotective effect of FGL without interfering with the antitumor effect of DOX and documented the molecular underpinning that FGL increased the deacetylation of PGC-1α by deacetylase SIRT1 through promoting the expression of SIRT1, and concurrently increased the expression of PGC-1α, to facilitate PGC-1α-mediated MB and FAO.

## Data Availability

The datasets presented in this study can be found in online repositories. The names of the repository/repositories and accession number(s) can be found below: https://www.ncbi.nlm.nih.gov/sra; PRJNA765675.
