# Supplementary material for: Ferruginol Restores SIRT1-PGC-1α-Mediated Mitochondrial Biogenesis and Fatty Acid Oxidation for the Treatment of DOX-Induced Cardiotoxicity
Source: Front Pharmacol. 2021 Nov 24;12:773834. doi: 10.3389/fphar.2021.773834 (PMC8652228; doi:10.3389/fphar.2021.773834)
Supplement: Supplementary file 1 [file DataSheet1.docx]

Supplementary Material

# Supplementary Data

## Materials

DOX was purchased from ApexBio Technology LLC (Houston, TX, United States). Enalapril (ENA) and FGL was purchased from Shanghai Yuanye Bio-Technology Co., Ltd. (Shanghai, China). Selisistat and SR-18292 were purchased from MedChemExpress (Monmouth Junction, NJ). Saline was purchased from SiYao Co., Ltd. (Shijiazhuang, China). A 2.5% glutaraldehyde solution and 5% BSA blocking buffer were purchased from Beijing Solaibao Technology Co., Ltd. (Beijing, China). Dulbecco’s modified Eagle’s medium (DMEM), foetal bovine serum (FBS), penicillin, streptomycin, phosphate-buffered saline (PBS), Cell Counting Kit-8 (CCK-8) and 4',6-diamidino-2-phenylindole (DAPI), Hoechst 33342 were purchased from Beijing BioDee Biotechnology Co., Ltd. (Beijing, China). Paraformaldehyde (4%) was purchased from Beijing Applygen Technology Inc. (Beijing, China). MitoTracker® Green FM was purchased from Cell Signaling Technology (Danvers, Massachusetts, USA). 2′,7′-dichlorofluorescin diacetate (DCFH-DA) was purchased from Sigma-Aldrich LLC (Shanghai, China). All other chemicals were purchased from commercial sources.

## Quantification of mitochondrial population and mtDNA copy number

Mitotracker Green staining was used to locate intracellular mitochondria, and then fluorescence microscopy was used to image. Total DNA was extracted using DNeasy Blood & Tissue kit (Qiagen, Hilden, Germany) following manufacturer's instruction. The number of mitochondrial DNA copy number in H9C2 cells was quantified by the short-range PCR (SRPCR) using FastStart Universal SYBR Green Master (Rox) and normalized to GAPDH. SRPCR primers amplified the mtDNA in 12S rRNA coding region, and its sequences were listed below: forward: 5’-ATGCACGATAGCTAAGACCCAA-3’; reverse: 5’-GCTGAATTAGCGAGAAGGGGTA-3’. GAPDH primer sequences: forward: 5’- GGAGAAACCTGCCAAGTATGA-3’; reverse: 5’- TTGAAGTCACAGGAGACAACC-3’.

# Supplementary tables

Table 1. Primers for quantitative real-time PCR

| Gene name | species | Forward or 5' primer | Reverse or 3' primer |
| --- | --- | --- | --- |
| Sirt1 | mouse | CGCTGTGGCAGATTGTTATTAA | TTGATCTGAAGTCAGGAATCCC |
| Sirt1 | rat | ACGCCTTATCCTCTAGTTCCTGTGG | CGGTCTGTCAGCATCATCTTCCAAG |
| Ppargc1a | mouse | GGATATACTTTACGCAGGTCGA | CGTCTGAGTTGGTATCTAGGTC |
| Ppargc1a | rat | ACAGAGAACAGAAACAGCAGCAGAG | GGGTCAGAGGAAGAGATAAAGTTGTTGG |
| Ppara | mouse | GAGCTGCAAGATTCAGAAGAAG | GAATCTTTCAGGTCGTGTTCAC |
| Ppara | rat | GCCAAGAGAATCCACGAAGCCTAC | TGTTGCTAGTCTTTCCTGCGAGTATG |
| Nrf1 | Mouse | GTTGCCCAAGTGAATTACTCTG | TCGTCTGGATGGTCATTTCAC |
| Nrf1 | rat | GTTGCCCAAGTGAATTACTCTG | TCGTCTGGATGGTCATTTCAC |
| Tfam | mouse | GTGAGCAAGTATAAAGAGCAGC | CTGAACGAGGTCTTTTTGGTTT |
| Tfam | rat | ACGCCTAAAGAAGAAAGCACAAATCAAG | TGACTCATCCTTAGCCTCCTGGAAG |
| Cd36 | mouse | CTTTGAAAGAACTCTTGTGGGG | GTCTGTGCCATTAATCATGTCG |
| Cd36 | rat | AAGCAGAAATGTTCAGAAACCAAGTGAC | TCCAACACCAAGTAAGACCATCTCAAC |
| Cpt2 | mouse | TGTCTTTGATGTCCTCGATCAA | TCGGTTCTCACTGGTCAAATAA |
| Cpt2 | rat | GAGCCTACCTGGTCAACGCATATC | AGCCTTGGTGTCAGTAAAGAGTTCATC |
| Lpl | mouse | CCTGATGACGCTGATTTTGTAG | CAATGAAGAGATGAATGGAGCG |
| Lpl | rat | ACTCTGTGTCTAACTGCCACTTCAAC | CTCATACATTCCTGTCACCGTCCATC |

Table 2. The antibodies used in this paper.

| Antibodies | Companies |
| --- | --- |
| Anti-acetyl Lysine | Ab190479; Abcam; United States |
| Anti-SIRT1 | 13161-1-AP; Proteintech; United States |
| Anti-NRF1 | CST46743; Cell Signaling Technology; United States |
| Anti-TFAM | 22586-1-AP; Proteintech; United States |
| Anti-CPT2 | 26555-1-AP; Proteintech; United States |
| Anti-CD36 | CST14347; Cell Signaling Technology; United States |
| Anti-PPARα | 15540-1-AP; Proteintech; United States |
| Anti- PGC-1α | Bs-1832R; Bioss; United States |
| Anti-PGC-1α | Sc-518025; Santa Cruz Biotechnology; United States |
